# Thermodynamic and Transport Properties of Equilibrium Debye Plasmas

**DOI:** 10.3390/e22020237

**Published:** 2020-02-20

**Authors:** Gianpiero Colonna, Annarita Laricchiuta

**Affiliations:** CNR Istituto per la Scienza e Tecnologia dei Plasmi (ISTP) Bari, via Amendola 122/D, 70126 Bari, Italy

**Keywords:** Debye plasmas, thermodynamics, pressure-ionization, electrical conductivity

## Abstract

The thermodynamic and transport properties of weakly non-ideal, high-density partially ionized hydrogen plasma are investigated, accounting for quantum effects due to the change in the energy spectrum of atomic hydrogen when the electron–proton interaction is considered embedded in the surrounding particles. The complexity of the rigorous approach led to the development of simplified models, able to include the neighbor-effects on the isolated system while remaining consistent with the traditional thermodynamic approach. High-density conditions have been simulated assuming particle interactions described by a screened Coulomb potential.

## 1. Introduction

The development of new technologies and experimental techniques has triggered intensive theoretical studies on modeling spatially confined quantum systems [1,2] and also extreme-high-pressure plasmas [3] like in stellar envelopes [4]. The thermodynamics of high-density hydrogen plasmas has been deeply investigated [5,6,7,8,9], due to the necessity of properly accounting for the effects of the multi-body interaction and in principle requiring the reformulation of the statistical mechanics in terms of a global Hamiltonian for the whole gas, instead of the usual separable form of non-interacting chemical species characterized through internal and translational partition functions. The non-ideality also affects the transport properties and in the case of dense, non-ideal, weakly-ionized Debye hydrogen plasma, the electrical conductivity in the non-metal-to-metal transition region at 150 GPa has been measured [10].

The investigation of the thermodynamic and transport properties of highly-dense hydrogen (and its isotopes) and helium plasmas is in fact relevant to many different fields, from astrophysics, for applications to low mass stars and giant planets [11], to inertial confinement fusion for the understanding of the ignition phase. Moreover, hot dense hydrogen and deuterium plasmas can be generated in a laboratory with shock compression, allowing the experimental accurate determination of the molecular-to-atomic transition along the principal Hugoniot to be compared with theoretical first-principle results [12].

It is also worth noting that atomic properties (level ensemble, electrical properties, static polarizability and hyperpolarizability [13,14,15] and optical oscillator strengths [16]) and the dynamics of collisions (electron impact excitation and ionization [17], symmetric charge exchange [18,19,20]) change in high-density regimes and are the subject in recent years of an intense investigation focused on the atomic hydrogen system.

In this paper, the thermodynamic properties and the electrical conductivity of weakly non-ideal, high-density partially ionized hydrogen plasma are investigated, accounting for quantum effects due to the change in the energy spectrum of atomic hydrogen when the electron-proton interaction is considered embedded in the surrounding particles. High-density conditions were simulated assuming atomic hydrogen described by a static screened Coulomb potential.

The Debye-Hückel or Yukawa potential, derived from the linearization of the exponential in the Poisson–Boltzmann equation [21,22], is considered suitable for the description of weakly-coupled plasmas, i.e., when the coupling parameter $\Gamma =1/(ak_{B}T_{e})\leq 1$, where $a={\left[3/\left(4\pi N_{e}\right)\right]}^{1/3}$ and $N_{e}$ is the free electron density, and has been used in the literature for the estimation of the effects on collision processes [17,19,23,24,25,26,27]. The conditions explored in the present paper, the electron density ranging from 10${}^{16}$ to 10${}^{23}$ cm${}^{-3}$ and the temperature from 10${}^{4}$ to 5 10${}^{4}$ K, are compatible with weak coupling up to $n_{e}$=10${}^{22}$, while for higher densities the value of Γ is greater than the unity and in principle would require a quantum approach to properly treat the interaction in these strongly-coupled plasmas. In fact, the chemical picture of the interaction offered by the Yukawa potential fails in a strongly correlated quantum regime, where other effects need to be accounted for, such as the ion-ion correlation, the electron exchange, the consistent statistics for electrons and therefore the accurate ab initial molecular dynamics method has to be resorted to [28,29,30]. Another important issue in both weakly- and strongly-coupled plasmas and neglected in this paper is the dynamical nature of screening, affecting the interaction potential between electrons and ions, and in turn, the transport properties of the plasma and the dynamics of elastic and reactive collisions [21,31,32]. In fact, plasma density fluctuations, due to inter-particle correlation in dense plasmas, produce time-dependent effects in the interaction of electrons and ions, due to the polarization induced by the electron on the surrounding plasma particles, that critically depends on the ratio between the electron velocity and its thermal velocity. The effect of dynamic screening on scattering processes in weakly-coupled plasmas has been investigated [25,33,34], showing that the use of static screening overestimates the shielding, therefore, we would expect an increase of the elastic transport cross-sections reducing the electrical and thermal conductivities.

## 2. Results

### 2.1. Thermodynamics

In weakly non-ideal, partially ionized Debye plasmas, the electron–proton interaction embedded in the surrounding particles can be adequately described by the Yukawa potential, i.e., the static screened Coulomb potential (in atomic units), which is

(1) $$U\left(r\right)=-\frac{\exp(-r/\lambda_{D})}{r}$$

where

(2) $$\lambda_{D}=\sqrt{k_{B}T_{e}/\left(4\pi N_{e}\right)}$$

is the Debye length, $k_{B}$ the Boltzmann constant, $T_{e}$ the electron temperature and $N_{e}$ the electron density, with severe confined conditions being related to small $\lambda_{D}$ values.

**Figure 1:**
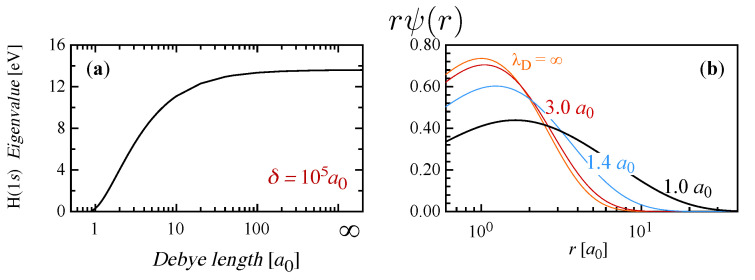
(**a**) Dependence of the ionization potential of atomic hydrogen on the Debye length (δ = 10${}^{5}$$a_{0}$). (**b**) Radial wavefunction of the H(1*s*) ground level for different screening conditions, from isolated atom ($\lambda_{D}$ = *∞*) to severe confinement corresponding to very low values of $\lambda_{D}$.

The atomic hydrogen levels have been calculated by discretization of the radial differential equation and for solving eigenvalues and eigenvectors for different screening conditions, from 2000 to 0.9 Bohr radii [$a_{0}$], so as to obtain a smooth description of the variation of level energy with the Debye length towards the critical transition to the continuum. In fact, the quantum effects act in modifying the H level structure and lead to a finite number of bound states [7,35]. As the screening increases, i.e., in very high-pressure regimes, the ground state moves towards the continuum, reducing the ionization potential, here estimated through the Koopman theorem, as shown in Figure 1a. Correspondingly, the radial wavefunction of the 1*s* level, displayed in Figure 1b, becomes more diffuse, describing a physical condition characterized by an electron loosely bound to the nucleus. The system of excited levels also move to the ionization limit, entering the continuum (Mott effect) [7], therefore, the number of bound levels progressively reduces as the Debye length decreases, up to a critical value of last existence of the only 1s state, below which bound states are not admitted and the plasma is fully ionized.

In the framework of statistical thermodynamics, the state functions are fully determined by the partition function of the system Q and the ionization equilibrium is governed by the Saha equation

(3) $$\frac{N_{H^{+}}N_{e}}{N_{H}}=\frac{2Q_{e}^{\mathrm{tr}}}{Q_{H}}\exp-\frac{I_{\mathrm{eff}}}{k_{B}T}$$

where $Q_{e}^{\mathrm{tr}}$ is the translational partition function and is derived for a plasma at pressure *p* in a continuum approximation, while the $Q_{H}$ is the internal partition function of atomic hydrogen.

(4) $$Q_{H}=2\sum_{n,\ell }^{n_{\max}}\left(2\ell +1\right)\exp\left[-\left(\varepsilon_{n,\ell }-\varepsilon_{1s}\right)/k_{B}T\right]$$

For ideal plasmas, the natural divergency of the internal partition function is avoided, truncating the summation in Equation (4) by using the cutoff criteria, i.e., the minimum value between the Fermi and the Griem cutoff [22]. In Debye plasmas, the finiteness of the number of atomic levels due to the screening presents the very attractive feature of a natural cutoff. In this case, the eigenvalues become dependent on the value of the Debye length that is consistent with the equilibrium in the plasma system, that is $\varepsilon_{n,\ell }^{\lambda_{D}}$ and in turn

(5) $$Q_{H}=2\sum_{n,\ell }^{n_{\lambda_{D}}}\left(2\ell +1\right)\exp\left[-\left(\varepsilon_{n,\ell }^{\lambda_{D}}-\varepsilon_{1s}^{\lambda_{D}}\right)/k_{B}T\right]$$

The mutual dependence of the Debye length and of the equilibrium value of the number density of electrons, $N_{e}$, makes the determination of $\lambda_{D}$ an iterative procedure that allows the self-consistency of the values characterizing the plasma at a given temperature and pressure. The non-ideal character of the plasma is usually accounted for, including the Debye-Hückel correction in the calculation of the lowering of the ionization energy [36]. This term for the hydrogen atom corresponds to the so-called self-energy shift, $\Delta =-e^{2}/\lambda_{D}$, thus leading to an effective value [7,22]$I_{\mathrm{eff}}=I_{0}+\Delta$, where $I_{0}$ is the ionization potential of the isolated, unperturbed hydrogen atom. However, the effect of the modification in the energy level scheme due to the screening also affects the internal partition function, producing an additional lowering that is incorporated in the internal partition function

(6) $$Q_{H}^{\prime }=Q_{H}\exp\left[-\left(\varepsilon_{1s}-\varepsilon_{1s}^{\lambda_{D}}\right)/k_{B}T\right]$$

It should be stressed that in the present paper, the free electrons are described through the classical Boltzmann statics, but for us to move to strongly non-ideal dense plasmas, the inclusion of the quantum Fermi statistics would be required [37,38,39].

**Figure 2:**
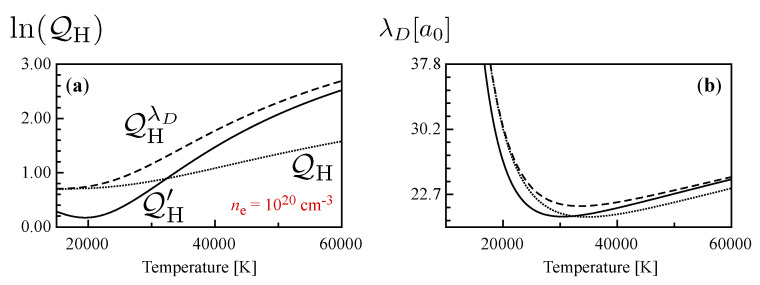
(**a**) Atomic hydrogen internal partition function as a function of temperature at $n_{e}$ = 10${}^{20}$ cm${}^{-3}$, calculated with the unperturbed levels with cut-off criteria, $Q_{H}$, including all the levels consistent with the Debye length in the plasma and accounting for the lowering of ionization potential, $Q_{H}^{\lambda_{D}}$, and considering the additional ionization lowering, $Q_{H}^{\prime }$. (**b**) Corresponding temperature behavior of the Debye length, self-consistently determined in the three cases.

In Figure 2a,b, the temperature dependence of the internal partition function and of the Debye length is self-consistently determined, following the notation adopted in the literature, for a specific value of the total electron density, $n_{e}\;=\;N_{e}+N_{H}$, i.e., electrons bound in an atomic system plus free electrons formed in ionization, $n_{e}$ = 10${}^{20}$ cm${}^{-3}$ are reported. The Debye length reported is actually calculated while also considering the shielding of ionic species and not only free electrons [22].

The results obtained using the eigenvalues for the unperturbed atom are compared with the partition functions calculated, accounting for the $\lambda_{D}$-dependent energy levels and of the additional lowering. The partition function in the case of unperturbed levels is actually lower than the values obtained by accounting for the actual levels, as already shown in the literature [40], in fact, the change in the energy-spacing of levels for a screened Coulomb potential allows a larger number of levels to be kept in the summation with respect to what was established with an external cutoff criterion. The inclusion of the additional lowering significantly affects the effective partition function, especially for temperature below 20,000 K. It is also worth noting that the Debye length is also affected in the three different cases attaining values of the order of tens of Bohrs.

The ionization degree $\alpha =N_{e}/\left(N_{e}+N_{H}\right)$ has been calculated at a constant total electron density, from 10${}^{16}$ to 10${}^{23}$ cm${}^{-3}$, over a wide temperature range [15,000–50,000 K]. For higher densities, the theoretical framework is no longer able to deal with the non-ideal effects in the presence of strongly-coupled plasmas and different approaches need to be considered [41].

**Figure 3:**
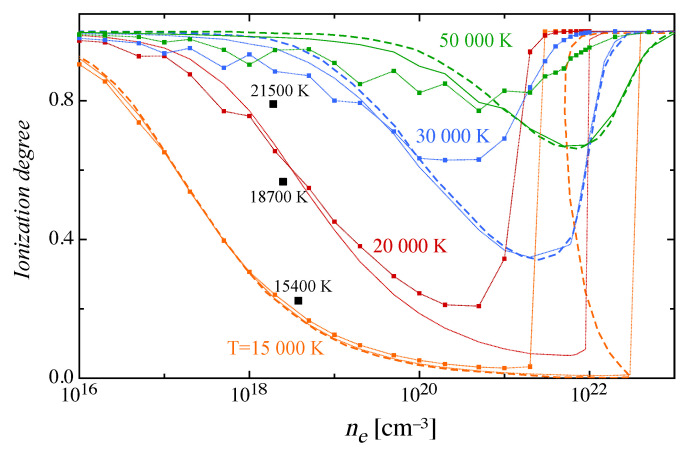
Isotherms of the ionization degree of atomic hydrogen plasma as a function of total electron density in the plasma $n_{e}$, obtained neglecting (dotted lines) and including (markers and lines) the effect of electronic levels, compared with theoretical results in the literature (dashed lines) [7]. Experimental results for a hydrogen arc at a pressure of 10 atm [42] are also reported (squares).

The isotherms are shown in Figure 3, and exhibit the phenomenon of pressure ionization [7,22], i.e., the rapid increase of α in the high-density regime. Contrary to what is expected in ideal plasmas, where the pressure (or density) increase produces a temperature shift in the ionization equilibrium, thus retarding its onset, the non-ideal quantum effects favor the ionization process and produce the observed increase of α merging to the fully ionized case in the limit of high temperature or very-high densities. The results obtained in this work considering only the Debye-Hückel correction to the ionization potential compare well with those reported in the literature [7]. The isotherm at 15,000 K presents a critical behavior around 2 × 10${}^{22}$ cm${}^{-3}$ that produces an ultra-fast transition to the full ionization and corresponds to the condition of lowest values for the Debye length and to the disappearance of any bound state for the atomic hydrogen. In the same figure, the isotherms are calculated including the effect of additional lowering of the ionization potential, due to the non-ideal effects on the electronic atomic structure, and these show a more pronounced pressure ionization.

Inspection of the isotherms clearly shows the presence of oscillations, more pronounced in the case of additional lowering. These oscillations are due to the fact that the internal partition function $Q_{H}$ has a non-regular behavior with the total electron density due to the induced modification in the atomic internal level structure, that introduces discontinuities. This behavior is mirrored on the equilibrium constant $K_{P}$ that shows a non-regular increase with the density differently from the pressure that increases rapidly and thus producing, as a combined effect, the oscillations in the molar fractions of species and in the ionization degree, representing an ultimate result of the Mott effect of bound levels transitioning to the continuum.

In Figure 3 the ionization degree derived from experiments in a hydrogen arc at a pressure of 10 atm [42] for three different temperature values, approximately corresponding to a total electron density of 10${}^{18}$ cm${}^{-3}$, are also reported, showing a satisfactory agreement with the theoretically predicted values. Unfortunately, there is no available experimental data that could validate the results at higher densities, that is where the non-ideal phenomenon manifests itself.

**Figure 4:**
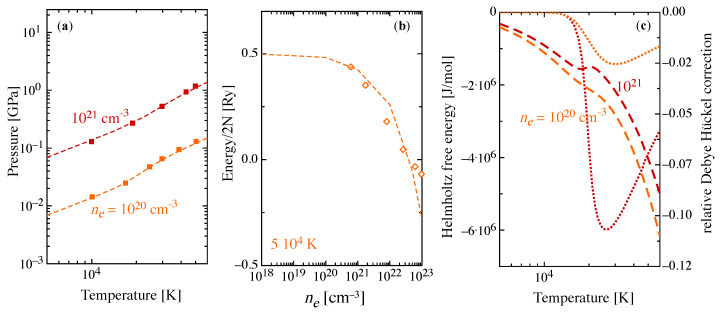
(**a**) Pressure isochors of a hydrogen plasma as a function of temperature for two different values of the total electron density (dashed lines) compared to results in the literature (closed squares) [41]. (**b**) Internal energy of the atomic hydrogen plasma as a function of the total electron density at the temperature *T* = 5 × 10${}^{4}$ K (dashed line) compared with results obtained in path integral Monte Carlo (PIMC) simulation [43]. (**c**) Helmholtz free energy as a function of temperature for two different values of the total electron density (dashed lines) and corresponding relative Debye-Hückel corrections, ΔA/A (dotted lines).

Concerning the validity of the present approach with respect to theories that can handle the quantum physics of plasmas even in strongly coupled conditions, the results derived for the hydrogen plasma by using the direct fermionic path integral Monte Carlo (PIMC) method [41,43] are compared with present results in Figure 4a,b. In Figure 4c, the temperature behavior of the Helmholtz free energy is also reported for two values of the total electron density.

(7) $$A=-k_{B}T\sum_{s}N_{s}\left(\ln\frac{Q_{s}}{N_{s}}+1\right)-\frac{1}{12}k_{B}T\frac{V}{\pi \lambda_{D}^{3}}$$

where *V* is the gas volume, $N_{s}$ is the number of particles of the *s*-th species and the last term on the right-side of equation represents the Debye-Hückel correction, contributing not more than 11%.

The PIMC simulations allow for the estimation of the internal partition function from configurational integrals that simultaneously includes the different interactions among elementary particles in the atomic system (electrons and protons) in the frame of a physical picture. The pressure isochors (Figure 4a) for two density values are in very good agreement with PIMC simulation [41]. Figure 4b displays the internal energy of the hydrogen plasma as a function of $n_{e}$ for a selected value of the temperature and the comparison, limited to the upper value explored in this work, shows again a satisfactory agreement with the PIMC simulation.

### 2.2. Transport: The Electrical Conductivity

The effects of non-ideality on transport properties have been investigated in the frame of the Chapman-Enskog theory [44]. As is well-known, in this theory, the binary interactions are described through the collision integrals, and the non-ideal quantum effects producing a change in the internal level structure of atoms also significantly affects the quantities directly related to the transport cross-sections. However, in this paper we are focused on the effect of the change in the thermodynamic equilibrium, due to the accounting for the additional lowering, on electrical conductivity and therefore the collision integrals for *e*-H and H-H interactions are assumed to be unaffected, the corresponding screening-independent transport cross-sections taken from the literature [45], while charged-particle interactions, including electron-electron, modeled with accurate Debye-length-dependent collision integrals by Mason [46,47], recently fitted in a wide temperature range in [48].

**Figure 5:**
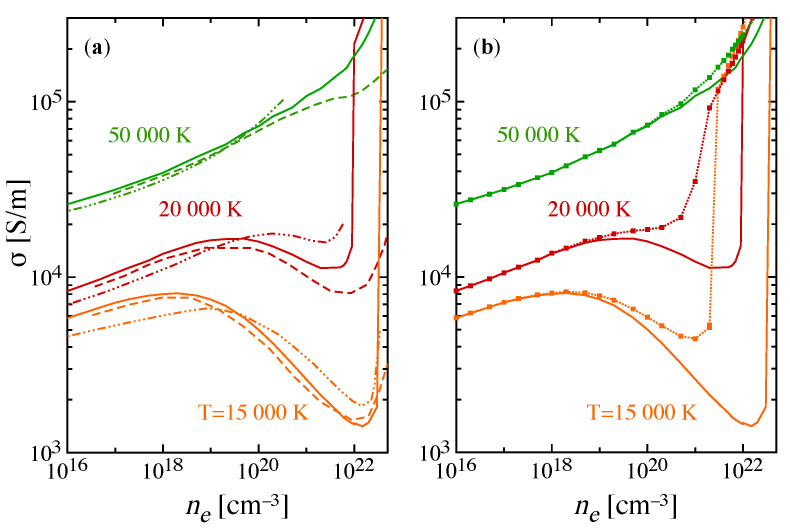
Electrical conductivity of an atomic hydrogen plasma for different temperatures as a function of the total electron density. The results (solid lines) obtained neglecting the additional lowering of ionization potential are compared with (**a**) data in literature (dashed lines) [49], (dashed-dotted lines) [37], and with (**b**) calculation including the additional lowering.

The electrical conductivity of the atomic hydrogen Debye plasma is displayed in Figure 5 as a function of the total electron density for three values of the temperature, considering the two cases, i.e., neglecting or accounting for the additional lowering of the ionization potential. In Figure 5a the σ exhibits a behavior with the increase of the screening in the plasma that is largely dependent on the electron density, and thus mirrors the phenomenon of the pressure ionization in Figure 3: The curves go through a minimum then merge to the fully ionized regime. This first series of results can be compared with the literature, obtained in the frame of different theories. In particular, in [49], the two-term Boltzmann equation is solved including in the collisional terms accurate elastic transport cross-sections for *e*-*e* and *e*-H interactions, re-evaluated so as to account for the additional screened Coulomb potential in the first Born approximation, while in [37] the linear response theory is used for transport. Both [37,49] neglect the contribution of excited levels in the atomic internal partition function, one dealing with the ground-state approximation and the second using the Planck-Larkin approach to avoid divergence which explains the satisfactory agreement found.

Accounting for the effect of the change in the level structure on the effective ionization of atomic hydrogen (Figure 5b) produces significant differences in the isotherms, especially at lower values of the temperature where the dip is pronounced, while the enhancement of the pressure ionization leads to a rapid increase towards the fully ionized case.

It is worth mentioning that for densities >0.1 g/cm${}^{-3}$ (for $n_{e}>6$ × 10${}^{22}$ cm${}^{-3}$) accurate electrical conductivity results have been obtained with finite-temperature density functional theory molecular dynamics (FT-DFT-MD) simulations [29]. Unfortunately, in this regime of strongly-coupled plasma, the assumption of Debye plasmas is no longer valid and an extension of the present approach to those densities are expected to be unreliable.

## 3. Conclusions

The non-ideal behavior of thermodynamic and transport properties of a partially-ionized, weakly-coupled atomic hydrogen plasma was investigated in the framework of the classical statistical approach and Chapman-Enskog theory, respectively. The approach adopted in literature accounts for the effects of surrounding plasma through the Debye-Hückel correction to the value of ionization potential and disregarding the change in the level ensemble of the H, that are considered in any conditions in those of the unperturbed isolated atom, limited in the internal partition function by different cutoff criteria. The accurate description of the level structure in different screening conditions also correspond to high-density regimes, which allows us to account for all the non-ideal effects on the equilibrium composition, i.e., the natural cutoff of bound levels in $Q_{H}$ and the further shift of the ground level to the continuum limit that determines an additional lowering of the effective ionization potential. The most significant result is represented by the emphasized phenomenon of pressure ionization, predicting a more rapid increase of the ionization degree with the total electron density in the whole temperature range considered. These results are expected to also impact the transport properties of the plasma, and in this work, the effect on the behavior of the electrical conductivity is demonstrated. The present results, though relying on the classical theoretical approaches, seem to compare well, at least for the density regime considered, with more accurate methods, reformulating the thermodynamics on the basis of a physical picture and accounting for the modification of transport cross-sections for the relevant interaction in the transport theory. In a future work, the contributions of Fermi statistics and dynamic screening will be investigated.
